# Prognostic utility of N-terminal pro B-type natriuretic peptide ratio in mixed aortic valve disease

**DOI:** 10.1136/openhrt-2023-002361

**Published:** 2023-07-19

**Authors:** Jérémy Bernard, Guillaume Jean, David Bienjonetti-Boudreau, Frédéric Jacques, Lionel Tastet, Erwan Salaun, Marie-Annick Clavel

**Affiliations:** 1 Cardiology, Institut universitaire de cardiologie et de pneumologie de Québec - Université Laval, Quebec, Quebec, Canada; 2 Cardiac Surgery, Institut universitaire de cardiologie et de pneumologie de Québec - Université Laval, Quebec, Quebec, Canada

**Keywords:** Aortic Valve Stenosis, Aortic Valve Insufficiency, Biomarkers, Outcome Assessment, Health Care

## Abstract

**Objective:**

We aimed to assess the incremental prognostic value of N-terminal-pro-B-type natriuretic peptide (Nt-proBNP) for risk stratification in mixed aortic valve disease (MAVD) patients.

**Methods:**

We included 556 (73±12 years, 37% women) consecutive patients with at least a moderate aortic stenosis (AS) or aortic regurgitation (AR) lesion with a concomitant AS or AR of any severity in whom Nt-proBNP was measured and expressed as its ratio (measured Nt-proBNP divided by the upper limit of normal Nt-proBNP for age and sex). The primary endpoint was all-cause mortality.

**Results:**

Baseline median Nt-proBNP ratio was 3.8 (IQR: 1.5–11.3), and the median follow-up was 5.6 years (4.8–6.1). Early aortic valve replacement (AVR) was performed within 3 months in 423 (76%) patients, while 133 (24%) remained initially under medical treatment. In comprehensive multivariable analyses, Nt-proBNP ratio was significantly associated with excess mortality (continuous variable: HR (95% CI): 1.24 (1.04 to 1.47), p=0.02; Nt-proBNP ratio ≥3: 2.41 (1.33 to 4.39), p=0.004). The independent prognostic value was also observed in patients with severe or non-severe AS/AR, and those treated by early-AVR (all p<0.04). Nt-proBNP ratio as continuous and dichotomic (≥3) variables showed incremental prognostic value (all net reclassification index >0.42, all p≤0.008). After early-AVR, Nt-proBNP ratio ≥3 was associated with higher 30-day mortality (9 (4%) vs 1 (0.5%), p=0.02).

**Conclusions:**

In this series of MAVD patients, Nt-proBNP ratio was a powerful predictor of early and long-term mortality, even in patients with both non-severe AS/AR. Moreover, early-AVR may be an option for patients with Nt-proBNP ratio ≥3. Further randomised studies are needed to validate this last point.

**WHAT IS ALREADY KNOWN ON THIS TOPIC**

- Management of mixed aortic valve disease (MAVD) patients remains challenging. A substantial proportion of MAVD patients treated with aortic valve replacement (AVR) present postoperative left ventricular (LV) dysfunction and poor outcomes, despite normal preoperative LV function. Patients with both moderate aortic stenosis (AS) and aortic regurgitation (AR) tend to present similar outcomes than isolated severe AS.

**WHAT THIS STUDY ADDS**

- Using a large population of MAVD patients with dosage of N-terminal-pro-B-type natriuretic peptide (Nt-proBNP) at the time on the index echocardiography, we demonstrated independent and incremental prognostic value of Nt-proBNP ratio (measured Nt-proBNP divided by the upper limit of normal Nt-proBNP for age and sex) in the management of these patients irrespectively of the treatment strategy (ie, AVR under 3 months or medical treatment) and severity of both lesions (ie, severe AS or AR or both non-severe lesions). The threshold of Nt-proBNP ratio ≥3 showed strong associations with increased 30 days and long-term mortality.

**HOW THIS STUDY MIGHT AFFECT RESEARCH, PRACTICE OR POLICY**

- These results suggest that Nt-proBNP would be an important tool to include in the management of MAVD patients. Early AVR may have an interest in patients with at least moderate AS or AR and concomitant AS or AR of any severity with a Nt-proBNP ratio ≥3, but further randomised trials need to address the benefits of early surgery in MAVD patients.

## Introduction

Mixed aortic valve disease (MAVD, ie, aortic valve stenosis (AS) and regurgitation (AR)) is a frequent condition.^1^ According to current American guidelines, MAVD patients should underwent intervention if peak aortic jet velocity (V_Peak_) is higher than 4 m/s or AR is severe and if they present symptoms or reduced left ventricular (LV) ejection fraction (<50%) (class I).^2^ Nonetheless, management of MAVD patients remains challenging, especially in patients with both non-severe AS and AR.^3 4^ Indeed, approximately 20% of MAVD patients and normal preoperative LV function develops LV dysfunction after aortic valve replacement (AVR),^5^ and delay in therapeutic intervention has been reported in MAVD patients.^6 7^ However, transcatheter or surgical AVR (TAVR or SAVR)^8–10^ have demonstrated survival benefit.^11^ Moreover, patients with concomitant non-severe AS and AR appear to have a similar outcome than patients with severe AS under medical management.^3^ Unfortunately, specific guidelines recommendations for these patients are lacking. Thus, robust and objective markers of outcomes are necessary to assist in surgical decision-making and timing in patients with MAVD. The use of natriuretic peptides provides additive prognostic value in patients with isolated AS^12 13^ or isolated AR,^14 15^ and was thoroughly validated in numerous studies including symptomatic or asymptomatic patients. We hypothesised that the use of N-terminal-pro-B-type natriuretic peptides (Nt-proBNP) in MAVD could further improve the risk stratification and optimise the timing of AVR. Hence, our objective was to assess the prognostic value and utility of Nt-proBNP ratio in a large and recent cohort of MAVD patients.

## Methods

### Study population

This is a single-centre cohort study that included all consecutive adult patients with at least moderate AS or AR (AS: assessed by aortic valve area (AVA) ≤1.50 cm^2^, peak aortic jet velocity (V_Peak_)≥3.0 m/s and mean transvalvular gradient (MG)≥20 mm Hg; AR: assessed by multiparameter approach) and a concomitant AS or AR of any severity who underwent comprehensive transthoracic Doppler-echocardiography (TTE) between January 2013 and February 2018 in the context of heart valve clinics at our institution (tertiary referral centre). Patients without measurement of Nt-proBNP during the same episode of care or with incomplete TTE were excluded. Other criteria of exclusions were: aortic valve (AV) disease related to active endocarditis, or rheumatic, or radiotherapy aetiology, and previous valvular intervention (online supplemental figure S1). All data were prospectively collected in an institutional database, but the analyses were performed retrospectively. The date of the first qualifying TTE during the selected duration of the study was defined as the baseline visit.

10.1136/openhrt-2023-002361.supp1 Supplementary data

### Clinical, laboratory and echocardiographic data

Baseline clinical and echocardiographic data are described in detail in online supplemental material. The aortic valve phenotype (ie, bicuspid or tricuspid) was carefully assessed by echocardiography.

Laboratory data were measured using automated techniques standardised with the Canadian reference laboratory. NT-proBNP was measured by an immunoassay on the Roche Elecsys 2010 Analyzer (similar to Mayo Clinic) during the entire study. The Nt-proBNP ratio was calculated as follows for each patient $Nt-proBNP-ratio=\;\frac{Measured\;plasma\;levels\;of\;Nt-proBNP\;}{Maximal\;normalvalues\;specific\;for\;age\;and\;sex\;}$,^12^ which were derived from Mayo Clinic laboratory (https://www.mayocliniclabs.com/test-catalog/overview/615897%23Clinical-and-Interpretive).

### Study endpoints

The primary endpoint was all-cause mortality regardless of treatment. Mortality was confirmed for all patients by a provincial institution (Institut de la statistique du Québec), thus follow-up was 100% complete. To maximise the interrogation of this central database, a list with multiple demographics (including first and last names, dates of birth and social security numbers) and a delay of 1 year between interrogation and last follow-up dates were used.

Initial treatment strategy was defined as the choice of management at study entry between early-AVR (≤3 months), either surgical or transcatheter approach, with or without concomitant interventions or initial conservative management (ie, no intervention or intervention >3 months). In the latter, survival analyses were censored at the time of AVR in those who underwent intervention after 3 months. This information was retrospectively retrieved from review of medical records. The decision for the type and timing of treatment was at the discretion of the treating physicians.

### Statistical analysis

Continuous data were tested for normality and homogeneity of variances with the Shapiro-Wilk and Levene tests, respectively, then expressed as mean±SD or median (IQR), and were compared between Nt-proBNP ratio groups (ie, ratio ≤1, ratio between 1 and 3, and ratio ≥3) using a one-way analysis of variance or Kruskal-Wallis, with multiple comparisons using the Bonferroni or C de Dunnett tests, as appropriate. Continuous variables who did not follow normal distributions, such as Nt-proBNP levels, were transformed using the natural logarithm (ln) for the regression models. Categorical variables are expressed as count and percentage and were compared using the χ^2^ test or Fisher’s exact test, as appropriate. Kaplan-Meier curves, univariable and multivariable Cox proportional hazards models were used to illustrate and compare the survival function. Patients were also separated according to MAVD patterns: severe AS/AR (V_Peak_≥4 m/s, MG≥40 mm Hg or ≥moderate-to-severe AR) and non-severe AS/AR (V_Peak_<4 m/s, MG<40 mm Hg and ≤moderate AR). Multivariable models were adjusted for clinically relevant variables and variables with a p<0.10 in univariate analysis (online supplemental table 1), all with considerations of avoiding overadjustment and collinearity. Results of the Cox models are presented as HR and 95% CIs. Subgroup analyses were performed to determine the association between Nt-proBNP ratio and mortality in each subgroup of MAVD patients. Net Reclassification Index (NRI) was used to determine the incremental value of Nt-proBNP ratio (ie, ≥3 vs <3) in predicting 1-year, 2-year and 5-year mortality, where patients lost to follow-up before the tested year were censored. A p<0.05 was considered statistically significant. Statistical analyses were performed with SPSS software (V.27, IBM) and Stata software (V.14.2, StataCorp).

## Results

### Characteristics of the study population

Among the 556 patients (mean age 73±12 years), 63% were male, 82% had systemic arterial hypertension, 53% had coronary artery disease (CAD), 14% had a bicuspid aortic valve and 38% were in New York Heart Association (NYHA) class III–IV. Median EuroSCORE II was 1.4 (0.9–2.5). Mean AVA was 1.27±0.93 cm^2^, mean V_Peak_ 4.2±0.8 m/s and mean MG 44±19 mm Hg. The respective combination of AS and AR severity is depicted in table 1. Median absolute value of Nt-proBNP was 515 (201–1778) ng/L and median Nt-proBNP ratio was 3.8 (1.5–11.3). Patients exhibit mean LV end-diastolic diameter (LVEDD) of 47±7 mm, LV end-systolic diameter (LVESD) of 31±9 mm, indexed LV mass of 109±32 g/m^2^ (table 1).

**Table 1:** Baseline characteristics of the study population

Variables	All patients (N=556)	Nt-proBNP ratio ≤1 (N=91; 16%)	Nt-proBNP ratio 1–3 (N=151; 27%)	Nt-proBNP ratio ≥3 (N=314; 57%)	P value
Clinical					
Age, years	73±12	68±11	71±11	75±11	<0.001*†
Male, n (%)	348 (63)	40 (44)	97 (64)	211 (67)	<0.001*‡
Body surface area, m²	1.8±0.3	1.9±0.2	1.9±0.2	1.8±0.3	0.12
Body mass index, kg/m²	28±5	29±6	28±5	27±6	0.14
NYHA class, n (%) (n=413)					0.08
I–II	257 (62)	51 (68)	79 (68)	127 (57)	
III–IV	156 (38)	24 (32)	37 (32)	95 (43)	
Hypertension, n (%)	452 (82)	68 (75)	117 (79)	267 (86)	0.01*
Diabetes, n (%)	148 (27)	24 (26)	36 (24)	88 (28)	0.66
Renal insufficiency, n (%)	170 (31)	8 (9)	32 (21)	130 (42)	<0.001*†
COPD, n (%)	89 (16)	12 (14)	18 (12)	59 (19)	0.10
Coronary artery disease, n (%)	292 (53)	38 (42)	74 (49)	180 (58)	0.01*
Atrial fibrillation, n (%)	199 (36)	15 (17)	42 (28)	142 (45)	<0.001*†
EuroSCORE II, %	1.4 (0.9–2.5)	1.0 (0.7–1.6)	1.1 (0.8–1.8)	1.7 (1.1–3.0)	<0.001*†
Blood sample data					
Haemoglobin, g/L	130 (117–140)	134 (125–146)	134 (122–144)	126 (111–138)	0.003*†
Creatinine, μmol/L	88 (75–107)	77 (66–88)	81 (73–96)	96 (81–121)	<0.001*†‡
Creatinine clearance, ml/min	69 (49–91)	89 (73–109)	77 (57–93)	58 (41–77)	<0.001*†‡
Nt-proBNP, ng/L	515 (201–1778)	84 (59–139)	242 (167–310)	1455 (707–3488)	–
Nt-proBNP ratio	3.8 (1.5–11.3)	0.7 (0.4–0.8)	1.8 (1.4–2.3)	10.0 (5.3–24.4)	–
Doppler echocardiography data					
Bicuspid aortic valve, n (%)	75 (14)	19 (21)	23 (15)	33 (11)	0.03*
Relative wall thickness	0.50±0.12	0.47±0.10	0.51±0.11	0.50±0.13	0.05
Indexed LV mass, g/m^2^	109±32	92±20	102±28	117±34	<0.001*†
Remodelling patterns (n=536)					<0.001*†
Normal, n (%)	80 (15)	22 (24)	21 (14)	37 (12)	
Concentric remodelling, n (%)	209 (39)	44 (49)	74 (50)	91 (30)	
Concentric hypertrophy, n (%)	154 (29)	13 (14)	36 (25)	105 (35)	
Eccentric hypertrophy, n (%)	54 (10)	5 (6)	7 (5)	42 (14)	
LVEDD, mm	47±7	45±6	45±6	48±8	0.01*†
LVESD, mm (n=478)	31±9	28±7	29±8	33±9	<0.001*†
Stroke volume, mL	125±96	121±79	139±101	119±97	0.14
LV ejection fraction, % (n=439)	56±11	61±5	61±6	53±13	0.006*†
LV ejection fraction <50%, n (%)	67 (12)	1 (1)	4 (3)	64 (25)	<0.001*†
Peak aortic jet velocity, m/s	4.2±0.8	4.0±0.7	4.3±0.7	4.3±0.9	0.03*†
Mean gradient, mm Hg	44±19	38±14	44±15	46±22	0.005*†‡
AVA, cm^2^	1.27±0.93	1.30±0.81	1.40±1.02	1.24±0.91	0.13
Moderate mitral regurgitation, n (%)	33 (6)	0 (0)	1 (1)	32 (11)	<0.001*†
≥Moderate TR, n (%) (n=470)	56 (10)	2 (3)	2 (2)	52 (19)	<0.001*†
sPAP, mm Hg (n=477)	35±16	29±9	30±12	39±17	<0.001*†
MAVD combination groups					**0.02**
Mild AS–mild AR	Excluded	Excluded	Excluded	Excluded	
Mild AS–moderate AR	31 (6)	5 (6)	6 (4)	20 (6)	
Mild AS–severe AR	9 (2)	0 (0)	4 (3)	5 (2)	
Moderate AS–mild AR	98 (18)	24 (26)	21 (14)	53 (17)	
Moderate AS–moderate AR	66 (12)	8 (9)	16 (11)	42 (13)	
Moderate AS–severe AR	13 (2)	4 (4)	2 (1)	7 (2)	
Severe AS–mild AR	176 (32)	35 (39)	57 (38)	84 (27)	
Severe AS–moderate AR	142 (26)	11 (12)	41 (27)	90 (29)	
Severe AS–severe AR	21 (4)	4 (4)	4 (3)	13 (4)	
MAVD severity groups					0.11
Severe AS or AR	361 (65)	54 (59)	108 (72)	199 (63)	
≤Moderate AS and AR	195 (35)	37 (41)	43 (28)	115 (37)	

Bold indicates statistical significance.

*Difference between Nt-proBNP≤1 and Nt-proBNP≥3.

†Difference between Nt-proBNP between 1 and 3 and Nt-proBNP≥3.

‡Difference between Nt-proBNP≤1 and Nt-proBNP between 1 and 3.

AR, aortic regurgitation; AS, aortic stenosis; AVA, aortic valve area; COPD, chronic obstructive pulmonary disease; EuroSCORE II, European System for Cardiac Operative Risk Evaluation II; LV, left ventricle; LVEDD, LV end diastolic diameter; LVESD, LV end systolic diameter; MAVD, mixed aortic valve disease; Nt-proBNP, N-terminal pro B-Type Natriuretic Peptide; NYHA, New York Heart Association; sPAP, systolic pulmonary artery pressure; TR, tricuspid regurgitation; V_peak_, peak aortic jet velocity.

Patients with elevated Nt-proBNP ratio were older, more often male and had more hypertension, renal insufficiency and CAD, thus more elevated EuroScore II (all p≤0.01; table 1), but less bicuspid aortic valve than patients with normal Nt-proBNP ratio (ie, ≤1). They also had higher LVEDD, LVESD and indexed LV mass, and lower LV ejection fraction despite in the normal range. Altogether AS and AR severity were comparable between groups, but patients with Nt-proBNP ratio ≥3 had more moderate mitral and tricuspid regurgitations (table 1). Of note, symptoms (NYHA functional class) do not discriminate patients with higher Nt-proBNP ratio (p=0.08; table 1) nor in early-AVR vs medical treatment (MT) (p=0.22; online supplemental table S2) and according to MAVD pattern (p=0.39; online supplemental table S3), which highlights the importance of Nt-proBNP in MAVD patients over symptoms.

### Association of Nt-proBNP ratio with mortality

During a median follow-up time of 5.6 years (IQR: 4.8–6.3), there were 97 (17%) deaths. Four hundred and twenty-three (76%) patients were referred for AVR within 3 months of the indexed echocardiography (317 (75%) SAVR, 86 (20%) TAVR, 10 (2.3%) Ross procedure, 10 (2.3%) Bentall procedure; 158 (37%) with concomitant myocardial revascularisation, and 32 (8%) aortic root/ascending aorta replacement), at a median time of 0.8 (0.2–1.8) months. Among the 133 (24%) patients initially under MT, 71 (53%) patients underwent AVR after 3 months, while 62 (47%) remained in MT during the entire follow-up (online supplemental figure S1).

**Figure 1:**
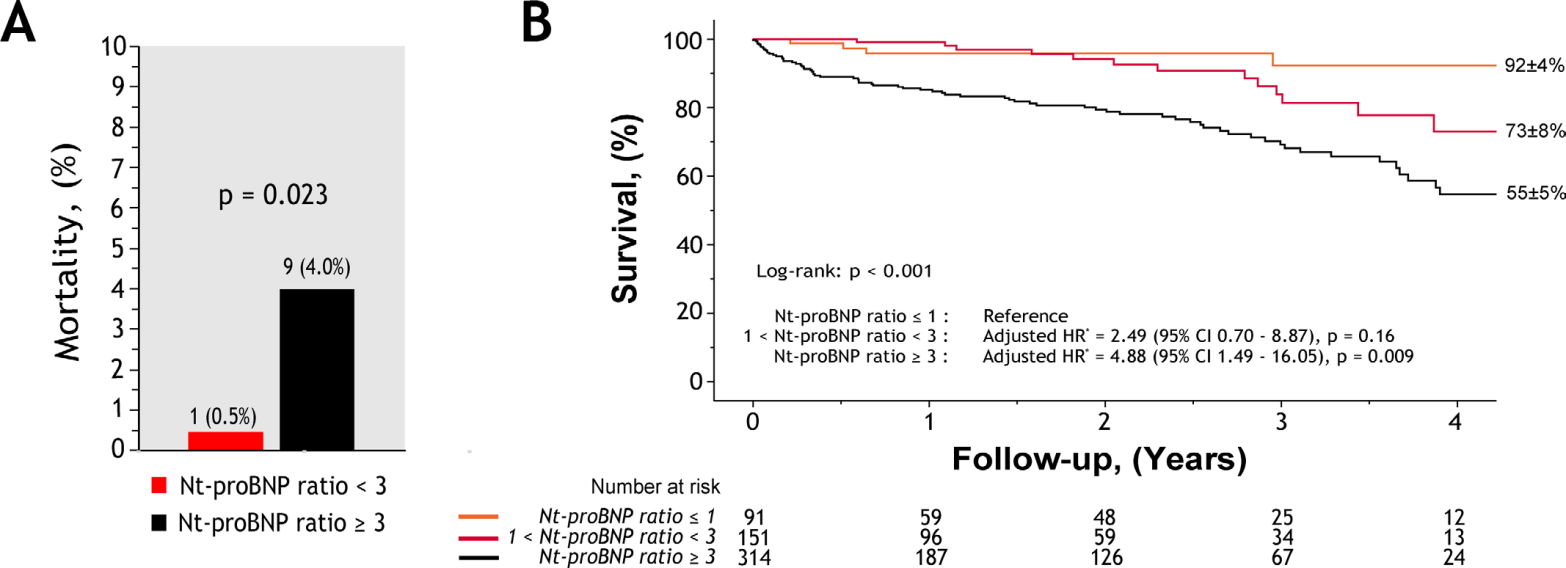
Association Nt-proBNP ratio with early and long-term outcomes in MAVD patients. (Left) rates of 30-day postoperative mortality after early-AVR according to patients with a Nt-proBNP ratio over or below 3. (Right) Kaplan-Meier curves of all-cause mortality according to Nt-proBNP ratio groups showing association with increased risk in MAVD patients with Nt-proBNP ratio ≥3. *Adjusted HR for EuroSCORE II, atrial fibrillation, natural logarithm of patient’s haemoglobin, LV end-diastolic diameter, V_peak_ and aortic valve intervention as a time-dependant variable, obtained by Cox multivariable analysis. AVR, aortic valve replacement; MAVD, mixed aortic valve disease; Nt-proBNP, N-terminal pro B-type natriuretic peptide.

Continuous and dichotomic Nt-proBNP ratio were associated with higher mortality (all p≤0.001), and there was a tendency toward significance for the 1<Nt-proBNP ratio <3 group (p=0.11) (table 2, figure 1). After comprehensive adjustment for EuroSCORE II, atrial fibrillation, ln(haemoglobin), LVEDD, V_peak_ and aortic valve intervention (as time-dependant variable), continuous Nt-proBNP ratio and Nt-proBNP ratio ≥3 remained independently associated with increased mortality (respectively, 1.23 (95% CI 1.02 to 1.50), p=0.03; 1.26 (95% CI 1.05 to 1.51), p=0.01; table 2).

**Table 2:** Associations of NT-proBNP with increased risk of all-cause mortality

Variables	All-cause mortality (97 events)					
			Model 1*		Model 2†	
	Univariable analysis		Multivariable analysis		Multivariable analysis	
	HR (95% CI)	P value	HR (95% CI)	P value	HR (95% CI)	P value
Ln Nt-proBNP	**1.62 (1.42 to 1.83**)	**<0.001**	**1.23 (1.02 to 1.50**)	**0.03**	**1.39 (1.04 to 1.86**)	**0.02**
Ln Nt-proBNP ratio	**1.61 (1.41 to 1.82**)	**<0.001**	**1.26 (1.05 to 1.51**)	**0.01**	**1.40 (1.04 to 1.87**)	**0.02**
Normal Nt-proBNP ratio (ie, ≤1)	Reference		Reference		Reference	
1<Nt-proBNP ratio <3	2.49 (0.82 to 7.56)	0.11	2.49 (0.70 to 8.87)	0.16	3.34 (0.68 to 16.40)	0.14
Nt-proBNP ratio ≥3	**7.20 (2.63 to 19.68**)	**<0.001**	**4.88 (1.49 to 16.05**)	**0.009**	**4.95 (1.08 to 22.65**)	**0.04**
Nt-proBNP ratio <3	Reference		Reference		Reference	
Nt-proBNP ratio ≥3	**3.85 (2.30 to 6.42**)	**<0.001**	**2.35 (1.37 to 4.61**)	**0.003**	2.07 (0.93 to 4.62)	0.07

Bold indicates statistical significance.

*Adjusted for EuroSCORE II, atrial fibrillation, ln haemoglobin, LVEDD, V_peak_ and aortic valve intervention as a time-dependant variable.

†Adjusted for age, sex, NYHA class, atrial fibrillation, COPD, CAD, renal insufficiency, ln haemoglobin, LVEDD, LV ejection fraction, V_peak_ and aortic valve intervention as a time-dependant variable.

CAD, coronary artery disease; COPD, chronic obstructive pulmonary disease; EuroSCORE II, European System for Cardiac Operative Risk Evaluation II; Ln, natural logarithm; LV, left ventricle; LVEDD, LV end diastolic diameter; Nt-proBNP, N-terminal pro B-Type Natriuretic Peptide; NYHA, New York Heart Association; V_peak_, peak aortic jet velocity.

### Association of Nt-proBNP ratio with mortality according to treatment strategy

Clinical and echocardiographic characteristics according to treatment strategy groups are presented in online supplemental table S2.

**Figure 2:**
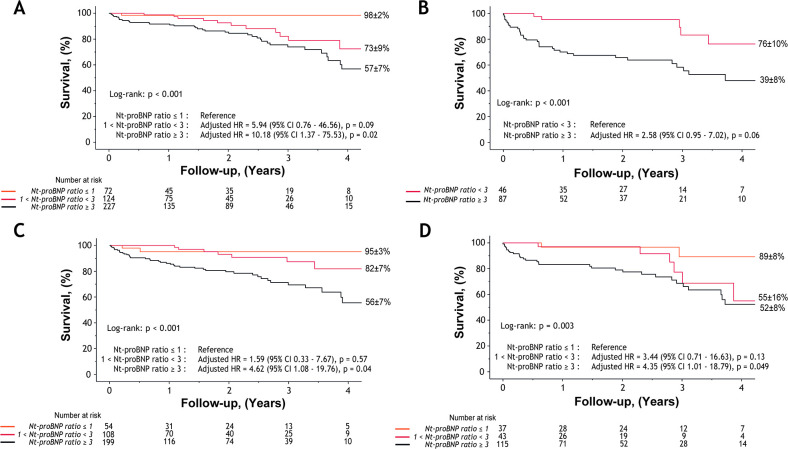
Survival in Nt-proBNP ratio groups according to treatment strategy and mixed aortic valve disease pattern. Kaplan-Meier curves of all-cause mortality according to Nt-proBNP ratio groups (ie, ratio ≤1, ratio 1–3 and ratio ≥3) in the subsets of patients (A) who underwent AVR within 3 months after echocardiography (n=423), (B) those who initially remained under medical treatment initially (n=133), (C) in patients with either severe AS or AR (n=361), and (D) in patients with ≤moderate AS and AR (n=195). Multivariate adjustments are as mentioned in table 3. AR, aortic regurgitation; AS, aortic stenosis; AVR, aortic valve replacement; Nt-proBNP, N-terminal pro B-Type natriuretic peptide.

Of the 423 patients who underwent early-AVR≤3 months, 53 (13%) died during follow-up. In this subset, Nt-proBNP ratio was strongly associated with long term all-cause mortality when analysed as continuous (1.51 (95% CI 1.25 to 1.82), p<0.001) and dichotomic (3.25 (95% CI 1.71 to 6.19), p<0.001) variables (online supplemental table S4; figure 2A). After comprehensive adjustment, Nt-proBNP ratio ≥3 remained associated to a ~2.5-fold increase in mortality (2.49 (95% CI 1.17 to 5.31), p=0.02; table 3). Moreover, Nt-proBNP ratio ≥3 was associated with higher 30-day postoperative mortality 9 (4%) vs 1 (0.5%), p=0.02; figure 1). Although limited in power, supplement analyses according to type of AVR revealed that continuous Nt-proBNP and its ratio (≥3) remained associated or tended to be in patients who received SAVR or TAVR (online supplemental table S5). For these analyses, patients treated with Bentall or Ross procedure were excluded (due to their small population).

**Table 3:** Multivariate associations of Nt-proBNP with all-cause mortality in subsets of patients

Variables	AVR under 3 months (N=423, 53 deaths)		Initial medical treatment (N=133, 44 deaths)		Severe AS or AR (N=361; 52 deaths)		Non-severe AS and AR (N=195; 45 deaths)	
	HR (95% CI)*	P value	HR (95% CI)*	P value	HR (95% CI)†	P value	HR (95% CI)†	P value
Ln Nt-proBNP	1.29 (0.98 to 1.70)	0.07	1.27 (0.97 to 1.65)	0.08	**1.36 (1.07–1.74**)	**0.02**	**1.38 (1.08–1.76**)	**0.01**
Ln Nt-proBNP ratio	**1.33 (1.01 to 1.74**)	**0.04**	1.27 (1.00 to 1.61)	0.052	**1.32 (1.05–1.65**)	**0.02**	**1.40 (1.12–1.75**)	**0.003**
Normal Nt-proBNP ratio (ie ≤1)	Reference		Reference		Reference		Reference	
1<Nt-proBNP ratio <3	5.94 (0.76 to 46.56)	0.09	0.49 (0.08 to 2.94)	0.43	1.59 (0.33–7.67)	0.57	3.44 (0.71–16.63)	0.13
Nt-proBNP ratio ≥3	**10.18 (1.37 to 75.53**)	**0.02**	1.80 (0.52 to 6.29)	0.36	**4.62 (1.08–19.76**)	**0.04**	**4.35 (1.01–18.77**)	**0.049**
Nt-proBNP ratio <3	Reference		Reference		Reference		Reference	
Nt-proBNP ratio ≥3	**2.49 (1.17 to 5.31**)	**0.02**	2.58 (0.95 to 7.02)	0.06	**3.27 (1.53–7.01**)	**0.002**	1.92 (0.88–4.20)	0.10

Bold indicates statistical significance.

*Adjusted for EuroSCORE II, ln haemoglobin level, LVEDD and V_peak_.

†Adjusted for EuroSCORE II, ln haemoglobin level and aortic valve intervention as a time-dependant variable.

AR, aortic regurgitation; AS, aortic stenosis; AVR, Aortic valve replacement; EuroSCORE II, European System for Cardiac Operative Risk Evaluation II; Ln, natural logarithm; LVEDD, LV end diastolic diameter; Nt-proBNP, N-terminal pro B-Type Natriuretic Peptide; V_peak_, peak aortic jet velocity.

In the initial MT group (n=133; 44 (33%) deaths), continuous Nt-proBNP ratio and Nt-proBNP ratio ≥3 were also associated with overall mortality (respectively, 1.61 (95% CI 1.34 to 1.93), p<0.001; 4.32 (95% CI 1.82 to 10.23), p<0.001; online supplemental table S4). After adjustment for EuroSCORE II, ln(haemoglobin), LVEDD and V_peak_, Nt-proBNP ratio ≥3 demonstrated a strong trend towards significant association (p=0.06) with a ~2.6-fold increase in mortality (figure 2B; table 3).

### Association of Nt-proBNP ratio with mortality according to MAVD pattern

Clinical and echocardiographic characteristics according to MAVD severity groups are presented in online supplemental table S3. In both groups (severe AS and/or severe AR; n=361 and non-severe AS and AR; n=195), Nt-proBNP ratio as a continuous, categorical or dichotomic variable remains significantly or strongly tended to be associated with all-cause mortality (table 3, online supplemental table S4 and figure 2C,D).

Additional adjustment by bicuspid aortic valve and ≥moderate tricuspid regurgitation in multivariate Cox analyses display similar results in total population and all subsets.

### Incremental prognostic value of Nt-proBNP ratio

In NRI analyses, Nt-proBNP ratio ≥3 provided significant incremental value to predict 1-year, 2-year and 5-year mortality (respectively, 44, 59 and 92 events) over clinical risk factors including EuroSCORE II, haemoglobin, LVEDD and V_peak_ (all NRI>0.4199, all p<0.008; table 4). Similar results were observed for continuous Nt-proBNP ratio (table 4).

**Table 4:** Incremental prognostic value of Nt-proBNP ratio to predict the risk of all-cause mortality

	NRI (95% CI) – 1 year (44 events)	P value	NRI (95% CI) – 2 years (59 events)	P value	NRI (95% CI) – 5 years (92 events)	P value
EuroSCORE II, ln haemoglobin, LVEDD and V_peak_.	Referent		Referent		Referent	
Ln Nt-proBNP ratio (continuous variable)	0.6961 (0.3633 to 1.0289)	**<0.0001**	0.4452 (0.1580 to 0.7324)	**0.002**	0.3656 (0.1288 to 0.6024)	**0.002**
EuroSCORE II, ln haemoglobin, LVEDD and V_peak_.	Referent		Referent		Referent	
Nt-proBNP ratio ≥3	0.4626 (0.1298 to 0.7954)	**0.005**	0.4794 (0.1922 to 0.7666)	**0.008**	0.4199 (0.1831 to 0.6567)	**0.0004**

Bold indicates statistical significance.

EuroSCORE II, European System for Cardiac Operative Risk Evaluation II; Ln, natural logarithm; LVEDD, LV end diastolic diameter; NRI, Net Reclassification Index; Nt-proBNP, N-terminal pro B-Type Natriuretic Peptide; V_peak_, peak aortic jet velocity.

### Interaction between BNP ratio and subgroups of MAVD patients

Only a trend for interaction of age with Nt-proBNP ratio ≥3 in regard to mortality was observed in subgroups analysis (p for interaction ≥0.07; online supplemental figure 2A). Nt-proBNP ratio as a continuous variable had a similar impact in all subgroups (online supplemental figure 2B).

## Discussion

The main findings of this study are that (1) Nt-proBNP ratio is strongly associated with all-cause mortality both in patients who underwent AVR, those with initial MT, and particularly in patients with combined non-severe AV lesions; (2) Nt-proBNP ratio provided significant incremental prognostic value beyond the traditional risk factors and clinical risk score to predict 1-year, 2-year and 5-year mortality; (3) Patients with Nt-proBNP ratio ≥3 are at high risk of early postoperative and long-term mortality. These findings suggest that Nt-proBNP ratio should be included as an additive clinical tool to enhance risk stratification in MAVD patients and that AVR in these patients may be reasonable before reaching a Nt-proBNP ratio ≥3.

### MAVD patients’ integrative evaluation

Management of patients with MAVD (ie, combined AS and AR) remains challenging in terms of intervention timing and risk stratification.^3 16 17^ Recent studies showed poor postoperative outcomes with high occurrence of adverse events despite normal preoperative LV function.^6 7^ Current management of MAVD patients is mainly driven by the guideline’s recommendations of the predominant valve disease but remains controversial, especially in the presence of both moderate AS and AR.^18^ In addition to the severity of the valve impairment, the assessment of the valvular-related global repercussion is essential to accomplish with objective markers, such as natriuretic peptides. The concomitant presence of volume and pressure overload brings challenges in the assessment of cardiac remodelling, ventricular function, and thus identification of the optimal timing for intervention,^19^ as numerous MAVD patients develop symptoms and/or LV dysfunction before reaching intervention cut-offs for either isolated AR or AS.^6 20^ The presence of a concentric remodelling or hypertrophy with a relative wall thickness over 0.42, an elevated LV filing pressure, and a decreased global longitudinal strain have been associated with poorer prognosis, but needs to be further validated in prospective studies.^21^

### Usefulness of the Nt-proBNP ratio in MAVD for risk stratification

BNP or Nt-proBNP were thoroughly validated in various isolated valvular heart diseases, either symptomatic or asymptomatic.^12 14 22 23^ Recently, Onishi *et al* demonstrated in a small MAVD population (ie, 81 patients) that BNP levels are independently associated with the endpoint of all-cause mortality, heart-failure hospitalisation and AVR.^24^ Nevertheless, as BNP or Nt-proBNP level increase with age and in women, and their elevation is lower in valvular heart diseases than heart failure, the use of the clinical activation ratio (ie, over the maximal expected value for age, sex and assay) has the advantage to take into account interpatient variability.^25^ In this study, median absolute Nt-proBNP value and its ratio were higher than what is observed in isolated AS or isolated AR, reflecting the presence of both volume and pressure overload.

In this study, continuous and dichotomised Nt-proBNP ratio independently and incrementally predicted survival. Increase in Nt-proBNP ratio demonstrated a dose–response relationship with an increase mortality in Nt-proBNP ratio ≥3 group superior than in Nt-proBNP ratio between 1 and 3 group. Moreover, patients with NT-proBNP≥3 have a substantial excess in mortality in all subsets. In the subset of patients who underwent AVR within 3 months, patients with preoperative Nt-proBNP ratio ≥3 had higher 30-day postoperative (4%) and long-term (43% at 4 years; ~2.5-fold increase) mortality. These patients may have irreversible preoperative subclinical LV dysfunction with myocardial fibrosis, which limits the potential improvement postoperatively. These results suggest that AVR in MAVD patients may be reasonable before reaching a Nt-proBNP ratio ≥3, even in combined non-severe lesions. Indeed, the latter had a ~1.9-fold increase in mortality with a strong trend after multivariable analysis when Nt-proBNP ratio was ≥3.

Finally, no sex-specific interaction in association between Nt-proBNP ratio and overall mortality has been found in this study. However, there is a clinical importance to normalise the absolute value for age, sex and each specific assay (ie, calculating the patient-specific Nt-proBNP ratio), as the absolute values increase with age and female sex.^25^

### Clinical implications

Altogether, the data presented in the study demonstrate that a single Nt-proBNP ratio measurement in MAVD patients has considerable standalone and incremental value in determining prognosis, irrespectively of treatment strategy or AV lesions severity. Thus, Nt-proBNP ratio should be integrated in the clinical decision-making process of these patients. Nt-proBNP is a sensitive and powerful marker of myocardial stretch and early subclinical dysfunction both in isolated AR and AS,^26^ which amplify its importance in MAVD patients’ management. According to our results, AVR may be reasonable in patients with at least moderate AS or AR before Nt-proBNP ratio reach 3, especially if the concomitant lesion is also moderate. However, the benefits of early surgery based on Nt-proBNP ratio threshold of three in MAVD patients’ needs to be addressed in prospective studies.^27^

Our data undoubtedly shows that Nt-proBNP ratio could largely enhance risk stratification in MAVD patients, especially in patients with combined non-severe AS and AR. As a matter of fact, natriuretic peptides reflect mainly severity of heart failure and not severity of valvular diseases and can be influence by other factors such as presence of other valvular diseases and atrial fibrillation, per se.^28^ Nevertheless, AVR decision in these patients should be based not only on severity of valvular disease but on overall cardiac remodelling, including assessment of Nt-proBNP ratio, valve lesion and general patient’s health. In the era of cardiac damage staging classification, the conceptualisation of a scheme for MAVD patients which includes Nt-proBNP ratio comprised between 1 and 3 could further contribute to comprehensive risk stratification.^29^

### Study limitations and strengths

Although the clinical, echocardiographic and outcome data were prospectively collected, the present analysis is of retrospective nature, and is thus subject to inherent limitations related to such design. However, this study includes a ‘real-life’ population, in which the patients were monitored in the context of heart valve clinics. Therapeutic decisions were left to the discretion of the patient’s treating physician, which referred patients according to its overall clinical presentation based of symptoms, signs and balance of risk/benefit of AVR. Our group who initially remained on MT is relatively small, and thus results have limited statistical power. The exact reasons for which these patients were treated medically are lacking. Nevertheless, more than 30% of patients in this subset died in early follow-up, and continuous Nt-proBNP ratio and ≥3 presented a strong trend towards being associated with all-cause mortality. One potential criticism could be that this cohort does not include high prevalence of severe AR. However, management of MAVD patients is mainly challenging in patients with non-severe AR/AS, and we demonstrated that Nt-proBNP was also independently associated with mortality in this subset. Reference Nt-proBNP values can differ between laboratories, but as they were dosed according to the Canadian reference laboratories guidelines, values remain highly accurate. We did not assess changes in Nt-proBNP ratio before and after AVR nor serial values in MT patients to test the potential additional prognostic value in risk assessment, but further studies should investigate these points.

## Conclusion

In this series of patients with MAVD, Nt-proBNP ratio is a powerful and incremental predictor of early postoperative and long-term mortality, both in patients who underwent AVR within 3 months, those initial under MT, and in combined non-severe AV lesions. These findings suggest that Nt-proBNP ratio is an additive clinical parameter to enhance risk stratification in MAVD patients and that AVR may be reasonable before reaching a Nt-proBNP ratio ≥3.

## Data Availability

No data are available. Data cannot be transmitted outside our institution due to a new Quebec provincial law.

## References

[1] Andell P, Li X, Martinsson A, et al. Epidemiology of valvular heart disease in a Swedish nationwide hospital-based register study. Heart 2017;103:1696–703. 10.1136/heartjnl-2016-310894 28432156 PMC5749343

[2] Otto CM, Nishimura RA, Bonow RO, et al. 2020 ACC/AHA guideline for the management of patients with valvular heart disease: executive summary: A report of the American college of cardiology/American heart Association joint committee on clinical practice guidelines. J Am Coll Cardiol 2021;77:450–500. 10.1016/j.jacc.2020.11.035 33342587

[3] Unger P, Clavel M-A. Mixed aortic valve disease: A diagnostic challenge, a Prognostic threat. Structural Heart 2020;4:468–74. 10.1080/24748706.2020.1817643

[4] Rashedi N, Popović ZB, Stewart WJ, et al. Outcomes of asymptomatic adults with combined aortic stenosis and regurgitation. J Am Soc Echocardiogr 2014;27:829–37. 10.1016/j.echo.2014.04.013 24874975

[5] Egbe AC, Warnes CA. Predictor of left ventricular dysfunction after aortic valve replacement in mixed aortic valve disease. Int J Cardiol 2017;228:511–7. 10.1016/j.ijcard.2016.11.237 27875727

[6] Egbe AC, Poterucha JT, Warnes CA. Mixed aortic valve disease: Midterm outcome and predictors of adverse events. Eur Heart J 2016;37:2671–8. 10.1093/eurheartj/ehw079 26994155

[7] Egbe AC, Luis SA, Padang R, et al. Outcomes in moderate mixed aortic valve disease: is it time for a paradigm shift? J Am Coll Cardiol 2016;67:2321–9. 10.1016/j.jacc.2016.03.509 27199054

[8] Chahine J, Kadri AN, Gajulapalli RD, et al. Outcomes of Transcatheter aortic valve replacement in mixed aortic valve disease. JACC Cardiovasc Interv 2019;12:2299–306. 10.1016/j.jcin.2019.06.020 31678084

[9] Abdelghani M, Cavalcante R, Miyazaki Y, et al. Transcatheter aortic valve implantation for mixed versus pure Stenotic aortic valve disease. EuroIntervention 2017;13:1157–65. 10.4244/EIJ-D-17-00328 28691910

[10] Philip JL, Zens T, Lozonschi L, et al. Outcomes of surgical aortic valve replacement for mixed aortic valve disease. J Thorac Dis 2018;10:4042–51. 10.21037/jtd.2018.06.128 30174847 PMC6106000

[11] Isaza N, Desai MY, Kapadia SR, et al. Long-term outcomes in patients with mixed aortic valve disease and preserved left ventricular ejection fraction. J Am Heart Assoc 2020;9:e014591. 10.1161/JAHA.119.014591 32204665 PMC7428636

[12] Clavel M-A, Malouf J, Michelena HI, et al. B-type natriuretic peptide clinical activation in aortic stenosis: impact on long-term survival. J Am Coll Cardiol 2014;63:2016–25. 10.1016/j.jacc.2014.02.581 24657652

[13] Nakatsuma K, Taniguchi T, Morimoto T, et al. n.d. B-type natriuretic peptide in patients with asymptomatic severe aortic stenosis. Heart:heartjnl–2018 10.1136/heartjnl-2018-313746 30530820

[14] Pizarro R, Bazzino OO, Oberti PF, et al. Prospective validation of the Prognostic usefulness of B-type natriuretic peptide in asymptomatic patients with chronic severe aortic regurgitation. J Am Coll Cardiol 2011;58:1705–14. 10.1016/j.jacc.2011.07.016 21982316

[15] Weber M, Hausen M, Arnold R, et al. Diagnostic and Prognostic value of N-terminal pro B-type natriuretic peptide (NT-proBNP) in patients with chronic aortic regurgitation. Int J Cardiol 2008;127:321–7. 10.1016/j.ijcard.2007.07.174 18055041

[16] Ong G, Pibarot P. Combined aortic stenosis and regurgitation: double the trouble. Heart 2019;105:1515–22. 10.1136/heartjnl-2017-312303 31142591

[17] Nedadur R, Belzile D, Farrell A, et al. Mixed aortic stenosis and regurgitation: a clinical conundrum. Heart 2023;109:264–75. 10.1136/heartjnl-2021-320501 35609962

[18] Unger P, Tribouilloy C. Aortic stenosis with other concomitant valvular disease: aortic regurgitation, mitral regurgitation, mitral stenosis, or Tricuspid regurgitation. Cardiol Clin 2020;38:33–46. 10.1016/j.ccl.2019.09.002 31753175

[19] Parker MW, Aurigemma GP. The simple arithmetic of mixed aortic valve disease: LVH+Volume Load= trouble. J Am Coll Cardiol 2016;67:2330–3. 10.1016/j.jacc.2016.03.549 27199055

[20] Byrd B, Baker M. Mixed aortic stenosis and regurgitation demands our attention. J Am Coll Cardiol 2013;61:1496–7. 10.1016/j.jacc.2013.01.029 23500258

[21] Egbe AC, Khan AR, Boler A, et al. Role of diastolic function indices in the risk stratification of patients with mixed aortic valve disease. Eur Heart J Cardiovasc Imaging 2018;19:668–74. 10.1093/ehjci/jex148 28655164

[22] Zhang B, Xu H, Zhang H, et al. Prognostic value of N-terminal pro–B-type natriuretic peptide in elderly patients with valvular heart disease. J Am Coll Cardiol 2020;75:1659–72. 10.1016/j.jacc.2020.02.031 32273031

[23] Hadziselimovic E, Greve AM, Sajadieh A, et al. Association of annual N-terminal pro-brain natriuretic peptide measurements with clinical events in patients with asymptomatic Nonsevere aortic stenosis: A post hoc Substudy of the SEAS trial. JAMA Cardiol 2022;7:435–44. 10.1001/jamacardio.2021.5916 35171199 PMC8851368

[24] Onishi H, Naganuma T, Izumo M, et al. Prognostic relevance of B-type natriuretic peptide in patients with moderate mixed aortic valve disease [ESC heart failure]. ESC Heart Fail 2022;9:2474–83. 10.1002/ehf2.13946 35543340 PMC9288736

[25] Redfield MM, Rodeheffer RJ, Jacobsen SJ, et al. Plasma brain natriuretic peptide concentration: impact of age and gender. J Am Coll Cardiol 2002;40:976–82. 10.1016/s0735-1097(02)02059-4 12225726

[26] Bergler-Klein J, Gyöngyösi M, Maurer G. The role of biomarkers in valvular heart disease: focus on natriuretic peptides. Can J Cardiol 2014;30:1027–34. 10.1016/j.cjca.2014.07.014 25151285

[27] Cremer PC, Rodriguez LL, Jaber WA. Early surgery for mixed aortic valve disease: A precocious or premature proposition? J Am Coll Cardiol 2016;68:1925–6. 10.1016/j.jacc.2016.06.074 27765199

[28] Ben-Dor I, Minha S, Barbash IM, et al. Correlation of brain natriuretic peptide levels in patients with severe aortic stenosis undergoing operative valve replacement or percutaneous Transcatheter intervention with clinical, echocardiographic, and hemodynamic factors and prognosis. Am J Cardiol 2013;112:574–9. 10.1016/j.amjcard.2013.04.023 23683951

[29] Tastet L, Généreux P, Bernard J, et al. The role of Extravalvular cardiac damage staging in aortic valve disease management. Can J Cardiol 2021;37:1004–15. 10.1016/j.cjca.2021.01.020 33539990

