# Supplementary material for: Prognostic utility of N-terminal pro B-type natriuretic peptide ratio in mixed aortic valve disease
Source: Open Heart. 2023 Jul 19;10(2):e002361. doi: 10.1136/openhrt-2023-002361 (PMC10357672; doi:10.1136/openhrt-2023-002361)
Supplement: Supplementary data [file openhrt-2023-002361supp001.pdf]

Supplemental Material for the following article

<u>Title:</u> Prognostic Utility of N-terminal pro B-type Natriuretic Peptides in Mixed Aortic Valve Disease

Jérémy Bernard, MSc; Guillaume Jean, MD; David Bienjonetti-Boudreau, MD; Frédéric Jacques, MD; Lionel Tastet, MSc; Erwan Salaun, MD, PhD; Marie-Annick Clavel, DVM, PhD

Table of Contents

Expanded Methods : Page 2
Supplemental Table 1: Page 3
Supplemental Table 2: Page 4-5
Supplemental Table 3: Page 6-7
Supplemental Table 4: Page 8
Supplemental Figure 1: Page 9
Supplemental Figure 2A: Page 10
Supplemental Figure 2B: Page 11

Bernard J, *et al. Open Heart* 2023; 10:e002361. doi: 10.1136/openhrt-2023-002361

EXPANDED METHODS

Clinical data

Baseline clinical data included age, sex, body surface area (BSA), body mass index, diagnosis of hypertension (patients receiving antihypertensive medications or having known but untreated hypertension [blood pressure≥140/90mmHg]), diabetes (patients receiving oral hypoglycemic or insulin medications, or, in the absence of such medications, having a fasting glucose ≥7mmol/L), coronary artery disease (history of myocardial infarction, significant coronary artery stenosis (i.e. >50%) on coronary angiography, and/or regional wall motion abnormality on echocardiogram), renal insufficiency (estimated glomerular filtration rate<60mL/min/1.73m^2), chronic obstructive pulmonary disease (COPD), and symptomatic status (New York Heart Association class).

Echocardiographic data

Transthoracic echocardiography was performed using commercially available ultrasound systems. Left ventricular (LV) diameters were measured according to current recommendations. LV mass was calculated and then indexed to BSA [1]. LV ejection fraction was calculated by the biplane Simpson method. The LV stroke volume was calculated using the LV outflow tract (LVOT) diameter (measured at the insertion of the aortic valve leaflets) and the LVOT velocity time integral. AS severity assessment included the evaluation of V_{Peak}, MG calculated with the simplified Bernoulli equation, and AVA calculated with the continuity equation [2]. AR, mitral and tricuspid regurgitation (TR) severity were assessed by integrated multiparameter approaches, as recommended by the current guidelines [3, 4]. Systolic pulmonary arterial pressure (sPAP) was derived from peak velocity of the regurgitant jet of TR and the estimated right atrial pressure [3].

Bernard J, *et al. Open Heart* 2023; 10:e002361. doi: 10.1136/openhrt-2023-002361

SUPPLEMENTAL TABLE 1: Multivariate Models Including the Predictors of Increased All-cause Mortality

Variables	All-Cause Mortality (97 events)					
			MODEL 1		MODEL 2	
	Univariable analysis		Multivariable analysis		Multivariable analysis	
	HR (95% CI)	p Value	HR (95% CI)	p Value	HR (95% CI)	p Value
Ln Nt-proBNP	**1.62 (1.42 - 1.83)**	**<0.001**	**1.23 (1.02 – 1.50)**	**0.03**	**1.39 (1.04 – 1.86)**	**0.02**
Age, 5 years increment	**1.29 (1.16 – 1.44)**	**<0.001**	**-**	**-**	1.11 (0.91 – 1.36)	0.30
Male sex	1.21 (0.80 – 1.83)	0.37	**-**	**-**	1.27 (0.63 – 2.57)	0.51
EuroSCORE II	**1.50 (1.35 - 1.67)**	**<0.001**	**1.38 (1.17 – 1.62)**	**<0.001**	**-**	**-**
NYHA 3-4 vs 1-2	1.17 (0.71 – 1.93)	0.53	**-**	**-**	1.15 (0.62 – 2.16)	0.65
Hypertension	1.18 (0.68 – 2.05)	0.56	**-**	**-**	**-**	**-**
Diabetes	1.16 (0.76 – 1.79)	0.49	**-**	**-**	**-**	**-**
COPD	**1.97 (1.26 – 3.07)**	**0.003**	**-**	**-**	1.94 (0.98 – 3.85)	0.06
Atrial fibrillation	**1.31 (0.92 – 1.85)**	**0.10**	1.16 (0.72 – 1.85)	0.53	1.07 (0.54 – 2.14)	0.84
Coronary artery disease	1.41 (0.93 – 2.13)	0.10	**-**	**-**	1.28 (0.64 – 2.57)	0.48
Renal insufficiency	**2.71 (1.81 – 4.07)**	**<0.001**	**-**	**-**	1.70 (0.89 – 3.27)	0.11
Ln Hemoglobin level	**0.29 (0.16 – 0.54)**	**<0.001**	0.53 (0.18 – 1.57)	0.25	0.80 (0.25 – 2.55)	0.71
V_{peak}, 0.2m/s increment	1.01 (0.96 – 1.06)	0.69	**1.06 (1.01 – 1.12)**	**0.03**	1.02 (0.94 – 1.11)	0.65
LVEDD, 5mm increment	1.14 (0.99 – 1.32)	0.06	**1.15 (1.01 – 1.31)**	**0.04**	0.98 (0.76 – 1.28)	0.91
Indexed SV, 5 ml/m^2 increment	0.98 (0.95 – 1.01)	0.19	**-**	**-**	**-**	**-**
LV ejection fraction, 5 % increment	**0.90 (0.82 – 0.98)**	**0.01**	**-**	**-**	0.88 (0.73 – 1.07)	0.20
Aortic valve intervention (as time-dependent variable)	**0.43 (0.27 - 0.68)**	**<0.001**	**0.43 (0.25 – 0.73)**	**0.002**	**0.45 (0.19 – 1.11)**	**0.08**

Bold indicates statistical significance. CI = confidence interval; COPD = chronic obstructive pulmonary disease; EuroSCORE II = European System for Cardiac Operative Risk Evaluation II; HR = hazard ratio; Ln = natural logarithm; LVEDD = LV end diastolic diameter; NYHA = New York Heart Association; SV = LV stroke volume; V_{peak} = Peak aortic jet velocity.

SUPPLEMENTAL TABLE 2: Baseline Characteristics According to Treatement Strategy

Variables	Early AVR (N = 423; 76%)	Initial MT (N = 133; 24%)
**Clinical**		
Age, years	72 ± 11	75 ± 12
Male, n(%)	271 (64)	77 (58)
BSA, m²	1.85 ± 0.25	1.82 ± 0.26
BMI, kg/m²	28 ± 5	29 ± 6
NYHA class, n(%) (n=429)		
I - II	195 (61)	62 (68)
III - IV	127 (39)	29 (32)
Hypertension, n(%)	345 (83)	107 (82)
Diabetes, n(%)	108 (26)	40 (30)
Renal insufficiency, n(%)	120 (29)	50 (38)
COPD, n(%)	60 (14)	29 (22)
CAD, n(%)	217 (52)	75 (58)
Atrial fibrillation, n(%)	146 (35)	53 (40)
EuroSCORE II, %	1.3 (0.9 – 2.2)	1.7 (1.1 – 3.1)
**Blood sample data**		
Hemoglobin, g/L	132 (118 – 141)	128 (115 – 136)
Creatinine, µmol/L	86 (74 – 103)	94 (77 – 125)
Creatinine clearance, ml/min	73 (51 – 93)	62 (42 – 84)
Nt-proBNP, ng/L	452 (191 – 1453)	826 (242 – 3639)
Nt-proBNP ratio	3.5 (1.5 – 10.0)	5.9 (1.8 – 20.4)
**Doppler echocardiography data**		
Bicuspid aortic valve, n(%)	12 (9)	63 (15)
Relative wall thickness	0.50 ± 0.12	0.48 ± 0.13
Indexed LV mass, g/m^2	109 ± 32	106 ± 32
Remodeling patterns (n=561)		
Normal, n(%)	57 (14)	22 (18)
Concentric remodeling, n(%)	158 (39)	51 (41)
Concentric hypertrophy, n(%)	130 (32)	24 (19)
Eccentric hypertrophy, n(%)	65 (16)	28 (22)
LVEDD, mm	47 ± 7	47 ± 8
LVESD, mm (n=478)	31 ± 8	31 ± 10
SV, mL	129 ± 97	113 ± 90
LV ejection fraction, % (n=459)	57 ± 10	55 ±12
LV ejection fraction <50%, n(%)	50 (15)	17 (17)
Peak aortic jet velocity, m/s	4.4 ± 0.8	3.9 ± 0.8
Mean gradient, mmHg	47 ± 19	36 ± 16
AVA, cm^2	1.27 ± 0.94	1.28 ± 0.90
Moderate mitral regurgitation, n(%)	22 (6)	11 (9)
≥moderate TR, n(%) (n=495)	38 (10)	18 (16)
SPAP, mmHg (n=477)	34 ± 15	39 ± 18
MAVD combination groups		
Mild AS – Mild AR	Excluded	Excluded
Mild AS – Moderate AR	12 (3)	19 (14)

Bernard J, *et al. Open Heart* 2023; 10:e002361. doi: 10.1136/openhrt-2023-002361

Mild AS – Severe AR	8 (2)	1 (1)
Moderate AS – Mild AR	63 (15)	35 (26)
Moderate AS – Moderate AR	45 (11)	21 (16)
Moderate AS – Severe AR	10 (2)	3 (2)
Severe AS – Mild AR	149 (35)	27 (20)
Severe AS – Moderate AR	117 (28)	25 (19)
Severe AS – Severe AR	19 (5)	2 (2)

Values are mean ± SD or median (interquartile range).
AR = aortic regurgitation; AS = aortic stenosis; AVA = aortic valve area; AVR = aortic valve replacement; BSA = body surface area; BMI = body mass index; CAD = coronary artery disease; COPD = chronic obstructive pulmonary disease; LV = left ventricle; LVESD = LV end systolic diameter; MI = myocardial infarction; Nt-proBNP = N-terminal pro B-Type Natriuretic Peptide; Abbreviations as in Supplemental Table 1.

SUPPLEMENTAL TABLE 3: Baseline Characteristics According to MAVD Severity Groups

Variables	Severe AS and/or AR; N = 361 ; 65%	≤Moderate AS and/or AR; N = 195; 35%
**Clinical**		
Age, years	71 ± 11	75 ± 11
Male, n(%)	231 (64)	117 (60)
BSA, m²	1.86 ± 0.26	1.83 ± 0.25
BMI, kg/m²	28 ± 5	28 ± 5
NYHA class, n(%) (n=429)		
I - II	165 (61)	92 (65)
III - IV	107 (39)	49 (35)
Hypertension, n(%)	291 (82)	161 (84)
Diabetes, n(%)	96 (27)	52 (27)
Renal insufficiency, n(%)	98 (27)	72 (37)
COPD, n(%)	57 (16)	32 (17)
CAD, n(%)	169 (47)	123 (64)
Atrial fibrillation, n(%)	121 (34)	78 (40)
EuroSCORE II, %	1.3 (0.9 – 2.2)	1.6 (1.1 – 2.9)
**Blood sample data**		
Hemoglobin, g/L	132 (116 – 142)	129 (117 – 138)
Creatinine, µmol/L	85 (74 – 102)	95 (76 – 116)
Creatinine clearance, ml/min	73 (52 – 93)	61 (41 – 84)
Nt-proBNP, ng/L	456 (202 – 1455)	642 (194 – 2768)
Nt-proBNP ratio	3.6 (1.6 – 10.3)	4.4 (1.5 – 13.3)
**Doppler echocardiography data**		
Bicuspid aortic valve, n(%)	54 (15)	21 (11)
Relative wall thickness	0.51 ± 0.12	0.47 ± 0.12
Indexed LV mass, g/m²	111 ± 32	103 ± 31
Remodeling patterns (n=561)		
Normal, n(%)	41 (12)	39 (21)
Concentric remodeling, n(%)	133 (38)	76 (40)
Concentric hypertrophy, n(%)	119 (34)	35 (19)
Eccentric hypertrophy, n(%)	54 (16)	39 (21)
LVEDD, mm	47 ± 7	47 ± 8
LVESD, mm (n=478)	31 ± 8	32 ± 9
SV, mL	136 ± 104	104 ± 74
LV ejection fraction, % (n=459)	58 ± 9	55 ± 13
LV ejection fraction <50%, n(%)	38 (13)	29 (20)
Peak aortic jet velocity, m/s	4.7 ± 0.7	3.4 ± 0.4
Mean gradient, mmHg	53 ± 18	28 ± 7
AVA, cm²	1.25 ± 0.94	1.31 ± 0.90
Moderate mitral regurgitation, n(%)	20 (6)	13 (7)
≥moderate TR, n(%) (n=495)	30 (10)	26 (16)
SPAP, mmHg (n=477)	35 ± 15	37 ± 17

Bernard J, *et al. Open Heart* 2023; 10:e002361. doi: 10.1136/openhrt-2023-002361

MAVD combination groups		
Mild AS – Mild AR	Excluded	Excluded
Mild AS – Moderate AR	0 (0)	31 (16)
Mild AS – Severe AR	9 (3)	0 (0)
Moderate AS – Mild AR	0 (0)	98 (50)
Moderate AS – Moderate AR	0 (0)	66 (34)
Moderate AS – Severe AR	13 (4)	0 (0)
Severe AS – Mild AR	176 (48)	0 (0)
Severe AS – Moderate AR	142 (39)	0 (0)
Severe AS – Severe AR	21 (6)	0 (0)

Values are mean ± SD or median (interquartile range).
Abbreviations as in Supplemental Table 1 and 2.

Bernard J, *et al. Open Heart* 2023; 10:e002361. doi: 10.1136/openhrt-2023-002361

<u>SUPPLEMENTAL TABLE 4:</u> Univariate Associations of Nt-proBNP with All-cause Mortality in Subsets of Patients

**Variables**	**AVR under 3 months** (N=423, 53 deaths)		**Initial medical treatment** (N=133, 44 deaths)		**Severe AS or AR** (N = 361; 52 deaths)		**Non-severe AS and AR** (N = 195; 45 deaths)	
	**HR (95% CI)**	**p Value**	**HR (95% CI)**	**p Value**	**HR (95% CI)**	**p Value**	**HR (95% CI)**	**p Value**
Ln Nt-proBNP	**1.50 (1.26 - 1.80)**	**<0.001**	**1.65 (1.36 – 1.99)**	**<0.001**	**1.58 (1.33 - 1.89)**	**<0.001**	**1.62 (1.34 – 1.95)**	**<0.001**
Ln Nt-proBNP ratio	**1.51 (1.25 - 1.82)**	**<0.001**	**1.61 (1.34 – 1.93)**	**<0.001**	**1.54 (1.29 - 1.85)**	**<0.001**	**1.64 (1.36 – 1.97)**	**<0.001**
Normal Nt-proBNP ratio (i.e. ≤1)	Reference		Reference		Reference		Reference	
1< Nt-proBNP ratio <3	7.35 (0.95 – 56.94)	0.06	0.86 (0.17 – 4.30)	0.86	1.80 (0.37 – 8.67)	0.46	3.81 (0.79 – 18.39)	0.10
Nt-proBNP ratio ≥3	**15.63 (2.15 – 113.69)**	**0.007**	**4.00 (1.23 – 13.01)**	**0.02**	**6.68 (1.61 – 27.74)**	**0.009**	**7.38 (1.78 – 30.68)**	**0.006**
Nt-proBNP ratio <3	Reference		Reference		Reference		Reference	
Nt-proBNP ratio ≥3	**3.25 (1.71 – 6.19)**	**<0.001**	**4.32 (1.82 – 10.23)**	**<0.001**	**4.37 (2.12 – 9.01)**	**<0.001**	**3.14 (1.51 – 6.53)**	**0.002**

Bold indicates statistical significance. Abbreviations as in Supplemental Table 1 and 2.

SUPPLEMENTAL TABLE 5: Association of Nt-proBNP with All-cause Mortality According to Type of AVR

Variables	SAVR (N = 366; 27 deaths)				TAVR (N = 105; 32 deaths)			
	Univariable analysis		Multivariable analysis*		Univariable analysis		Multivariable analysis*	
	HR (95% CI)	p Value	HR (95% CI)	p Value	HR (95% CI)	p Value	HR (95% CI)	p Value
Ln Nt-proBNP	**1.53 (1.17 - 1.99)**	**0.002**	1.33 (0.99 – 1.78)	0.06	1.20 (0.93 – 1.54)	0.16	1.22 (0.89 - 1.66)	0.22
Ln Nt-proBNP ratio	**1.52 (1.17 - 1.96)**	**0.001**	**1.35 (1.03 – 1.77)**	**0.03**	1.22 (0.95 – 1.58)	0.12	1.23 (0.92 - 1.65)	0.16
Normal Nt-proBNP ratio (i.e. ≤1)	Reference		Reference		Reference		Reference	
1< Nt-proBNP ratio <3	6.59 (0.81 – 53.70)	0.08	7.10 (0.87 – 58.09)	0.07	-	-	-	-
Nt-proBNP ratio ≥3	**9.96 (1.33 – 74.48)**	**0.03**	**8.45 (1.12 – 63.57)**	**0.04**	-	-	-	-
Nt-proBNP ratio <3	Reference		Reference		Reference		Reference	
Nt-proBNP ratio ≥3	**2.57 (1.12 – 5.87)**	**0.03**	2.11 (0.91 – 4.91)	0.08	**2.62 (1.00 – 6.87)**	**0.049**	2.57 (0.97 – 6.86)	0.06

Bold indicates statistical significance. SAVR = surgical aortic valve replacement; TAVR = transcatheter aortic valve replacement. Other abbreviations as in Table 3.
*Adjusted for EuroSCORE II.

Bernard J, *et al. Open Heart* 2023; 10:e002361. doi: 10.1136/openhrt-2023-002361

BMJ Publishing Group Limited (BMJ) disclaims all liability and responsibility arising from any reliance placed on this supplemental material which has been supplied by the author(s)

SUPPLEMENTAL FIGURE 1: Study Flow Chart

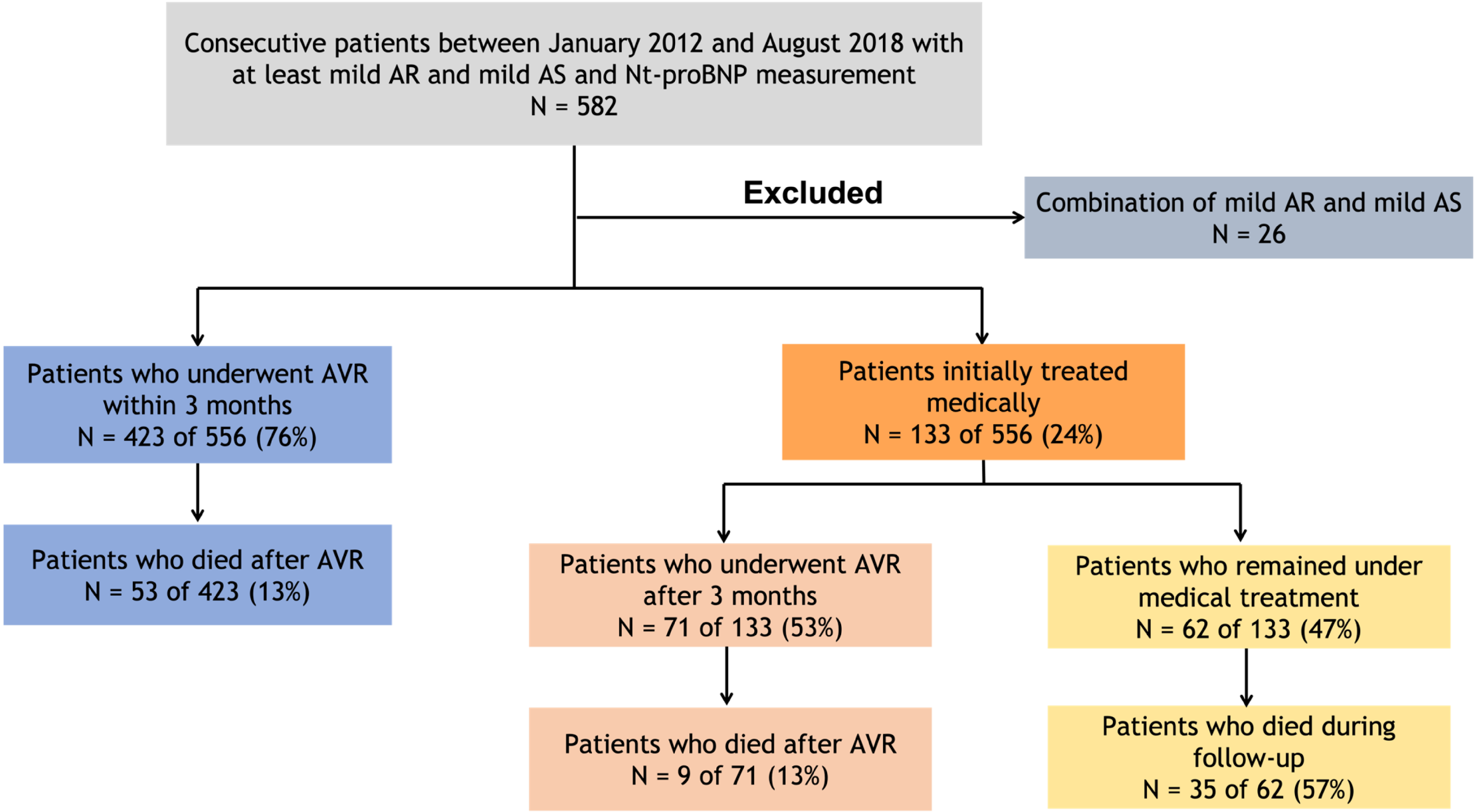

Caption: The figure shows the patients included in the study and the definition of treatment groups.
Legend: AR = aortic regurgitation; AS = aortic stenosis; AVR = aortic valve replacement; Nt-proBNP = N-terminal pro B-Type Natriuretic Peptide.

SUPPLEMENTAL FIGURE 2A: Subgroups Analysis of Survival for Nt-proBNP ratio ≥3

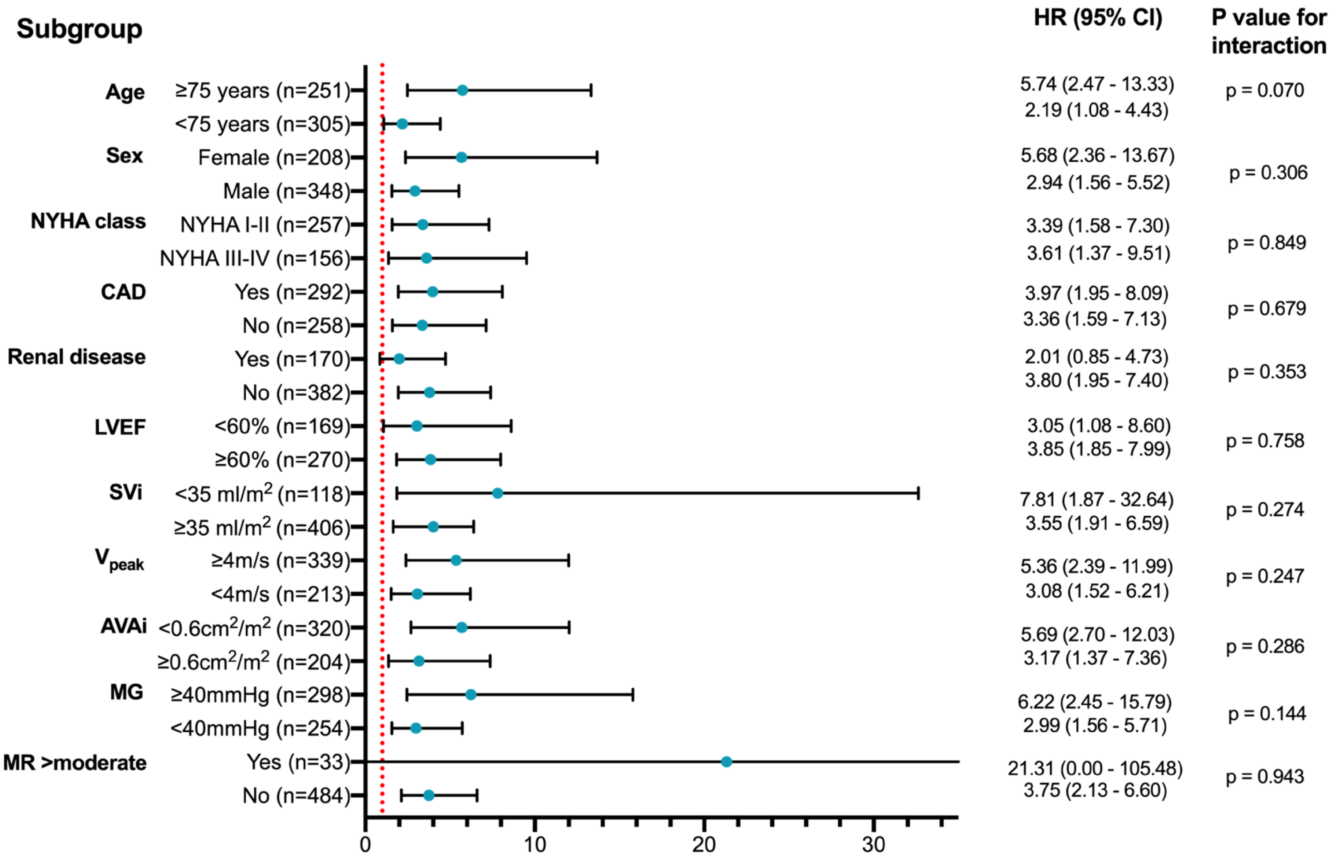

Caption: Nt-proBNP ratio ≥3 threshold and associated risk of all-cause mortality in various subgroups of MAVD patients. Hazard ratio (HR) and 95% confidence intervals (CI) for risk of mortality are presented for each subgroup. ***Legend:*** AVAi = aortic valve area index; CAD = coronary artery disease; COPD = chronic obstructive pulmonary disease; LVEF = left ventricular ejection fraction; NYHA = New York Heart Association; MG = mean gradient; MR = mitral regurgitation; SVi = LV stroke volume index; V_{peak} = Peak aortic jet velocity.

Bernard J, *et al. Open Heart* 2023; 10:e002361. doi: 10.1136/openhrt-2023-002361

SUPPLEMENTAL FIGURE 2B: Subgroups Analysis of Survival for Continuous Nt-proBNP ratio

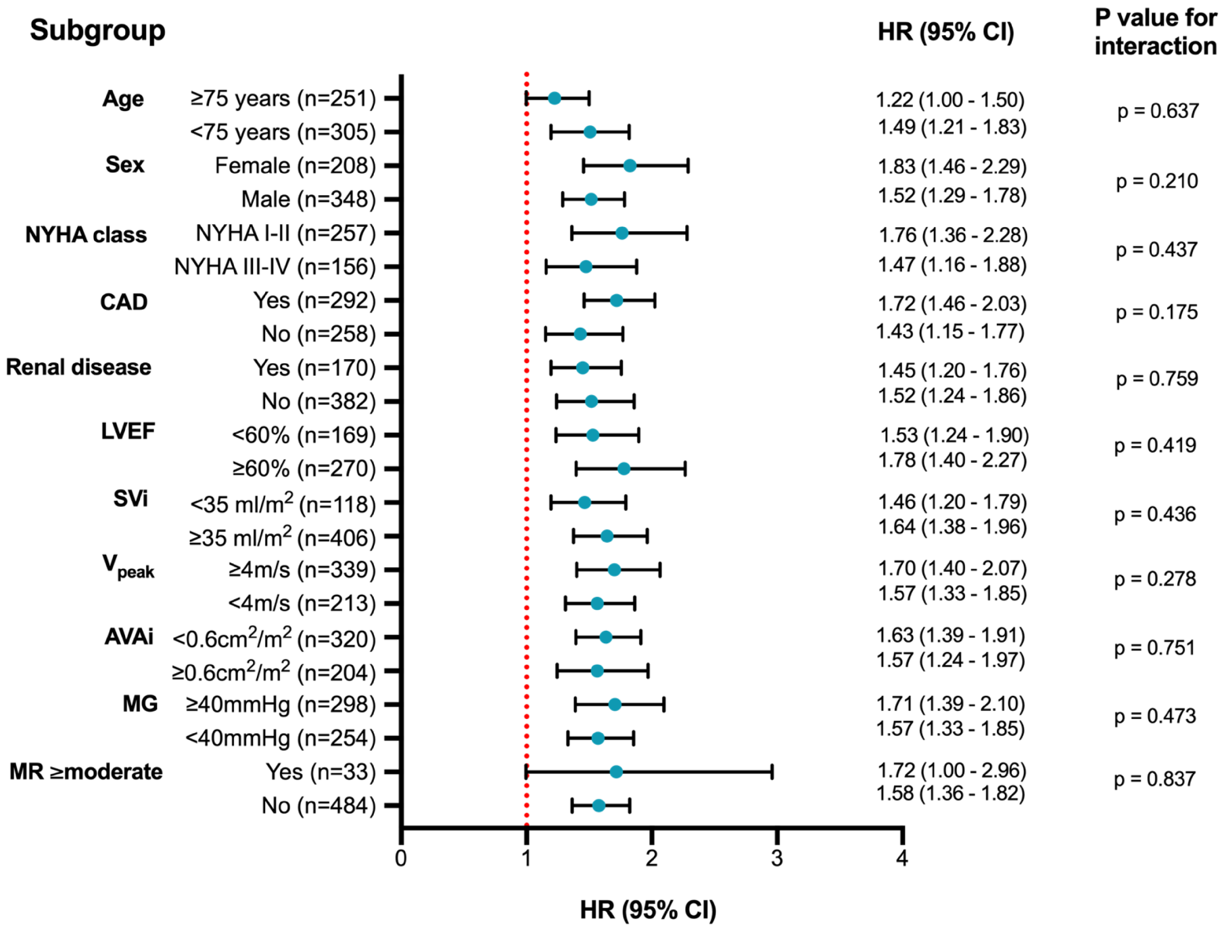

Caption: Continuous Nt-proBNP ratio and associated risk of all-cause mortality in various subgroups of MAVD patients. Hazard ratio (HR) and 95% confidence intervals (CI) for risk of mortality are presented for each subgroup. Abbreviations as in Supplemental Figure 2A.

Bernard J, *et al. Open Heart* 2023; 10:e002361. doi: 10.1136/openhrt-2023-002361

REFERENCES FOR SUPPLEMENTAL MATERIAL:

1 Lang RM, Badano LP, Mor-Avi V, et al. Recommendations for cardiac chamber quantification by echocardiography in adults: an update from the American Society of Echocardiography and the European Association of Cardiovascular Imaging. *Journal of the American Society of Echocardiography : official publication of the American Society of Echocardiography* 2015;**28**:1-39.e14.

2 Baumgartner H, Hung J, Bermejo J, et al. Recommendations on the echocardiographic assessment of aortic valve stenosis: A focused update from the European Association of Cardiovascular Imaging and the American Society of Echocardiography. *J Am Soc Echocardiogr* 2017;**30**:372-92.

3 Lancellotti P, Tribouilloy C, Hagendorff A, et al. Recommendations for the echocardiographic assessment of native valvular regurgitation: an executive summary from the European Association of Cardiovascular Imaging. *Eur Heart J Cardiovasc Imaging* 2013;**14**:611-44.

4 Zoghbi WA, Adams D, Bonow RO, et al. Recommendations for non invasive evaluation of native valvular regurgitation: A report from the American Society of Echocardiography developed in collaboration with the Society for Cardiovascular Magnetic Resonance. *J Am Soc Echocardiogr* 2017;**30**:303-71.
